# Inclusion of dietary nontoxic sulfur on growth performance, immune response, sulfur amino acid content and meat characteristics in growing-finishing pigs

**DOI:** 10.5713/ab.22.0418

**Published:** 2023-01-24

**Authors:** Hae Won Shin, Xing Hao Jin, Min Jin Gim, Yoo Yong Kim

**Affiliations:** 1 Department of Agricultural Biotechnology and Research Institute of Agriculture and Life Science, Seoul National University, Seoul 08826, Korea

**Keywords:** Growing-finishing Pigs, Growth Performance, Immune Response, Nontoxic Sulfur

## Abstract

**Objective:**

This experiment was conducted to evaluate the inclusion of dietary nontoxic sulfur (NTS) on growth performance, immune response, sulfur amino acid composition and meat characteristics in growing-finishing pigs.

**Methods:**

A total of 140 crossbred pigs ([Yorkshire×Landrace]×Duroc) with an average body weight of 34.73±0.66 kg were used for the 12-week feeding trial. Experimental pigs were allotted to one of 5 treatments in 4 replicates of 7 pigs per pen in a randomized complete block (RCB) design. The experimental treatments were as follows (0%, 0.1%, 0.2%, and 0.4% NTS levels): i) Control, corn soybean meal (SBM)-based diet; ii) NTS 0.1, basal diet + NTS 0.1%; iii) NTS 0.2, basal diet + NTS 0.2%; iv) NTS 0.4, basal diet + NTS 0.4%.

**Results:**

Body weight increased linearly as dietary NTS levels increased up to 0.2% (linear; p = 0.04) in the early finishing phase (9 weeks). During the whole experimental period, body weight and average daily gain linearly increased as the dietary NTS level increased in the diet (linear; both p = 0.01), but quadratic responses in body weight and average daily gain were observed with the addition of NTS 0.4% (quadratic, both p = 0.01). In the late finishing period, the IgG concentration increased linearly (linear; p = 0.01) as the dietary NTS level increased up to 4%. In the finishing period, a linear response was observed as a dietary NTS level was added (linear; p = 0.03), and supplementation with 0.2% NTS resulted in a higher methionine content than the other treatments (quadratic; p = 0.01). NST 0.2% had a lower value of thiobarbituric acid reactive substances (quadratic; p = 0.01).

**Conclusion:**

Consequently, supplementation with dietary NTS up to 0.2% could improve growth performance, amino acid composition in hair and meat antioxidation capacity.

## INTRODUCTION

Little attention has been given to the requirement and addition of sulfur in swine nutrition [1]. However, sulfur is an important component of sulfur amino acids and other organic compounds, such as methionine and cysteine, and it also plays an important role in their metabolism [2]. Therefore, many nutritionists are more concerned about sulfur amino acids rather than sulfur itself in swine nutrition [3]. Recently, many studies have been performed to indicate the main functions of sulfur, contributing to anti-inflammation, antioxidant and antibacterial abilities [4–6]. However, unprocessed sulfur or natural sulfur sources have high toxicity and generally induce side effects such as anorexia and weight loss in animals [7]. Hence, it is essential to remove the toxicity from natural sulfur, and detoxified sulfur might be used in livestock feed [8].

Studies in which dietary sulfur was used as a new feed additive were mostly focused on poultry nutrition and showed that supplemented dietary sulfur in poultry diets had positive effects on growth performance, meat quality and the strength and thickness of eggshell [9,10]. However, little information is available about using dietary sulfur in pig diets [11,12]. Kerr et al [13] reported that daily weight gain was decreased and the amount of sulfate-reducing bacteria and bacteria in feces were increased as increasing levels of inorganic sulfur (0.21% to 1.21%) were supplemented to a growing pig diet. Perez et al [14] also reported that weight gain was decreased when piglets fed an inorganic sulfur level increased from 0.2% to 0.6%. However, some previous studies by Cho et al [11] and Jang et al [15] showed positive effects on weight gain and feed consumption in pigs when dietary sulfur was provided to growing finishing pig diets. Upadhaya et al [16] also reported that body weight (BW) and weight gain increased linearly with increasing nontoxic sulfur (NTS) levels in the diet during finishing period. Recently, positive effects of dietary sulfur on pork quality, immune response and antioxidant ability were observed in pigs [17,18].

As shown above, growth performance and meat quality were improved when dietary sulfur was added to poultry feed. However, there is still a lack of published data on the effect of dietary NTS in growing-finishing pigs. Therefore, this experiment was conducted to verify the effects of dietary sulfur on growth performance, immune response, and meat characteristics in growing-finishing pigs.

## MATERIALS AND METHODS

### Experimental animals and management

All experimental procedures involving animals were conducted following the Animal Experimental Guidelines provided by the Seoul National University Institutional Animal Care and Use Committee (SNUIACUC; SNU-211213-5). A total of 140 crossbred pigs ([Yorkshire×Landrace] ×Duroc) with an average BW of 34.73±0.66 kg were allotted to one of five treatments considering sex and initial BW in 4 replications with 7 pigs (3 male pigs and 4 female pigs) per pen in a randomized complete block design.

Pigs were reared in growing-finishing (2.60×2.84 m) facilities for 12 weeks and supplied with a feeder and water nipple to provide feed and water *ad libitum* during experimental periods. The experimental period lasted 12 weeks and consisted of 4 phases: phase 1 was weeks 1 to 3, phase 2 was weeks 4 to 6, phase 3 was weeks 7 to 9 and phase 4 was weeks 10 to 12. Based on the collected data of BW and feed intake at the end of each phase, the average daily gain (ADG), average daily feed intake, and gain-to-feed (G:F) ratio were calculated step by step in each phase. Feed supply to all of the treatments was recorded each day, and waste feed left in the feeder was recorded at the end of each phase. Mortality or any health problems were not observed during the whole experimental period.

### Dietary treatments

The pigs in the four treatments were fed different levels of NTS, and the inclusion rates were 0%, 0.1%, 0.2%, and 0.4%. The addition of NTS containing more than 97% elemental sulfur used in the experiment was obtained from Nara Bio Co., Ltd. (Gunsan, Korea). Analyzed total sulfur (S) contents were as follows: i) Control, 1,600 mg/kg; ii) NTS 0.1, 2,760 mg/kg; iii) NTS 0.2, 3,550 mg/kg; iv) NTS 0.4, 5,840 mg/kg. All nutrients in the experimental diets met or exceeded the nutrient requirements of the NRC [1]. The formula and chemical composition of the experimental diet are presented in Table 1.

### Sample collection and analysis

Blood samples were taken from the jugular vein of six randomly selected pigs in each treatment to measure the immune response (IgA, IgG) when the BW was recorded. All blood samples were enclosed into serum tubes (SSTTM II Advance; BD Vacutainer, Becton Dickinson, Plymouth, UK). Collected blood samples were centrifuged for 15 min at 3,000 rpm at 4°C (5810R; Eppendorf, Hamburg, Germany). The serum was carefully transferred to 1.5 mL plastic tubes and stored at −20°C until analysis. The immunoglobulin G (IgG) and IgA concentrations were analyzed by enzyme-linked immunosorbent assay (ELISA) according to the manufacturer’s protocols (ELISA Starter Accessory Package, Pig IgG ELISA Quantitation Kit, Pig IgA ELISA Quantitation Kit; Bethyl, Montgomery, TX, USA).

Hair samples of approximately 2 to 5 g were collected from the dorsal midline from the white- and dark-haired pigs at 3-, 6-, 9-, and 12-week periods in each treatment pen. The hair was brushed before being clipped to remove adhered foreign material. The collected hair samples were obtained only from fully developed hair and from the site adjacent to the previous clipped area. For determination of the sulfur amino acid contents, including methionine and cysteine, a sample of 50 mg of hair was hydrolyzed for 65 min at 110°C in 1 mL of 6 N HCl (1% phenol) dissolved in 1 mL of 20 mM HCl, and 100 μL of this solution was diluted with water to 1,000 μL. The final solution was analyzed by high-performance liquid chromatography at the Institute of Agricultural Science, Chungnam National University.

### Carcass traits

At the end of the experiment, 4 pigs from each treatment group were selected and slaughtered at an average of 115.8± 1.05 kg for carcass analysis. Pork samples were collected from the nearby 10th rib on the right side of the carcass. After the chilling procedure, 30 minutes after slaughter was regarded as the initial time. The pH was measured at 0, 3, 6, 12, and 24 hours, and the meat color of the longissimus muscle was measured at an initial time and 24 hours after the initial time. The pH was measured using a pH meter (Model 720; Thermo Orion, Fullerton, CA, USA) and pork color was measured by CIE color L*, a*, and b* values using a CR300 (Minolta Camera Co., Osaka, Japan). Chemical analysis of pork samples was conducted by the AOAC method [19].

### Meat characteristics

The water-holding capacity (WHC) of pork was measured by the centrifuge method. Longissimus muscles were ground and sampled in a filter tube, heated in a water bath at 80°C for 20 min and centrifuged for 10 min at 2,000 rpm at 10°C (5810R; Eppendorf, Hamburg, Germany). To calculate the cooking loss, longissimus muscles were packed in a polyethylene bag and heated in a water bath until the core temperature reached 72°C and weighed before and after cooking. After heating, the samples were cored (1.27 cm in diameter) parallel to the muscle fiber, and the cores were used to measure the shear force using a Salter (Warner Barzler Shear, Norwood, MA, USA). The cooking loss, shear force, thiobarbituric acid reactive substances (TBARS) and WHC of pork were analyzed by animal origin food science, Seoul National University.

### Statistical analysis

All collected data were carried out by least squares mean comparisons and were evaluated with the general linear model procedure of SAS (SAS Institute Inc., Cary, NC, USA). Every pen was used as one unit in the feeding trial, and the individual pig was used as an experimental unit in immune response, sulfur amino acid composition, and pork quality. Orthogonal polynomial contrasts were performed to determine linear and quadratic effects of inclusion levels of dietary NTS. Statistical differences were considered highly significant differences at p<0.01, significant differences at p<0.05, and tendencies between p≥0.05 and p≤0.10.

## RESULTS AND DISCUSSION

### Growth performance

The effect of dietary NTS on growth performance is presented in Table 2. Body weight increased linearly as dietary NTS levels increased up to 0.2% (linear; p = 0.04) in the early finishing phase (9 weeks) and then decreased with a quadratic trend when a high level of NTS 0.4% was provided (quadratic; p = 0.08). Meanwhile, the ADG and G:F ratio decreased in the NTS 0.4% treatment compared with the other treatments (quadratic, p = 0.01 and p = 0.02, respectively). During the whole experimental period, BW, and ADG linearly increased as the dietary NTS level increased in the diet (linear, both p = 0.01), but quadratic responses in BW and ADG were observed in the addition of NTS 0.4% (quadratic, both p = 0.01).

In recent years, many studies on the supplementation of inorganic sulfur to animal feed have been conducted [17,20]. According to research by Kerr et al [13], the daily gain and daily feed intake linearly decreased as the inorganic sulfur level increased from 0.625% to 2.5% in the pig diet. Perez et al [14] also reported that increasing levels of inorganic sulfur in piglet diets linearly decreased ADG and feed intake as the inclusion rate increased from 0.2% to 0.6%. However, Thamaraikannan et al [21] stated that there was no significant difference in BW, daily weight gain, or feed intake when 10 ppm detoxified nano sulfur in the diet was fed to growing pigs for 10 weeks. In the current study, during the growing phase, increasing the level of dietary NTS did not affect the growth performance, but there was a linear improvement in BW by increasing the levels of NTS as well as the ADG in finishing periods, except under NTS 0.4% supplementation. Choudhury et al [22] indicated that inorganic sulfur could improve immunity and have an antibacterial function in the animal body. Therefore, treatment with 0.2% NTS had a positive effect on growth performance, and this result was associated with the immune response (Table 3), which showed that the IgG concentration linearly increased with the addition of dietary NTS. However, the high level of NTS 0.4% resulted in lower BW and weight gain compared to the other treatments due to toxicity. The main reason seems to be the toxin from dietary inorganic sulfur. Although dietary inorganic sulfur product was detoxified, the toxin was supposed to appear clearly as an increase in dietary NTS levels. Rather, excessive amounts of inorganic sulfur can cause negative effects on pigs. Moreover, there was no significant difference in growth performance during the growing phase, which also indicated that accumulated toxin in dietary sulfur additive would take a long time to negatively act on the growth of pigs.

### Immune response

The effect of dietary NTS levels on the immune response is presented in Table 3. There was no significant difference in IgA when pigs were fed different dietary NTS levels during the whole experimental period. In the late finishing period, the IgG concentration increased linearly (linear, p<0.01) as the dietary NTS level increased up to 4%, and then a quadratic trend was observed (quadratic; p = 0.08).

In general, IgA plays an important role in protecting mucosal surfaces when toxins, viruses, and bacteria enter or bind to a mucosal surface [23]. IgG accounts for the larger concentration of immunoglobulin in serum and extravascular spaces and is primarily involved in systemic defensive action [24]. In the late finishing period, the IgG concentration linearly increased because inorganic sulfur supplied in the feed was activated to combine with organic compounds in the animal body and then could provide a bioavailable sulfur source [25], and more sulfur-containing amino acids then accumulated in the body, which could indirectly enhance the immunity of growing-finishing pigs and improve the anti-inflammatory symptoms [26]. However, some reports have shown that NTS has antibacterial functions *in vivo* and *in vitro* [4,27], but it is not certain that dietary NTS could directly affect the production and secretion of immunoglobulin in swine. Therefore, the addition of NTS as a new additive to animal feed requires more research to explore the relationship between the immune system and the action of NTS.

### Sulfur amino acid content in hair

The effect of dietary NTS on sulfur amino acid content from pig hair is presented in Table 4. In the growing period, methionine content tended to increase linearly as the dietary NTS level increased up to 2% (linear; p = 0.08) and then decreased with a quadratic trend (quadratic; p = 0.06). In the finishing period, a linear response was observed as a dietary NTS level was added (linear; p = 0.03), and supplementation with 0.2% NTS resulted in a higher methionine content than the other treatments (quadratic; p = 0.01).

Dietary sulfur is the sulfur source of cysteine and methionine in nature [28]. Finkelstein and Mudd [29] reported that NTS could be activated to use as a source of sulfur to inhibit the decomposition of sulfur-containing amino acids and then improve the efficiency of protein synthesis when NTS was supplied to growing-finishing pigs. In the current study, there was no significant difference in cystine content from pig hair with supplying different levels of NTS, but methionine content increased linearly in growing-finishing stages and then decreased with a quadratic effect as the level of dietary NTS increased up to 4%.

### Carcass traits

The effect of dietary NTS on proximate analysis is presented in Table 5. In this study, an increased tendency was observed in moisture, and a declining trend was found in crude fat (p = 0.09 and p = 0.07, respectively). In addition, NST 0.2% had a lower value of TBARS (quadratic; p = 0.01).

Currently, many nutritionists have paid more attention to the application of dietary sulfur in livestock feed, which could increase the content of unsaturated fatty acids in pork and play an important role in lipid antioxidation [12,30,31]. Park et al [20] reported that fat content could decrease with an increase in dietary sulfur in broiler feed. Furthermore, higher water content and lower fat content were observed in ham meat when dietary sulfur was supplied to the fattened pig diet. This result was similar to the results of the current study. There was no significant difference in the physical and chemical properties of pork in terms of heating loss, shear force, and WHC. Heating loss is one of the indirect indicators of evaluating WHC, and there is a contrary relationship between the WHC and heating loss. Water-holding capacity is an important factor to measure pork quality [32]; in detail, high water hold capacity could improve pork quality. Lee et al [12] reported that when dietary sulfur was added to pig feed, the WHC was improved under low-temperature storage conditions. An analysis of TBARS is an important measurement to estimate lipid oxidation and rancidity or shelf life [31]. There was agreement with the results of Lee et al [12] and Kim et al [17], who demonstrated that dietary sulfur supplementation of pig feed could inhibit the oxidation of fat in pork. In addition, Mukwevho et al [33] also reported that the antioxidant capacity was improved by strongly eliminating free radicals when dietary sulfur was absorbed to produce sulfur compounds. Therefore, when NTS 0.2% was added to the feed diet in growing-finishing pigs, it could inhibit the rancidity of fatty acids and prolong the storage period.

### Meat characteristics

The pH change of pork is a very important factor in determining the pork’s freshness, tenderness, meat color and storage. It can affect the WHC, which has a strong influence on pork quality. Water-holding capacity determines both drip loss from raw pork and cooking loss during preparation. In this study, a linear increase (linear; p = 0.04) was found as dietary NTS increased to a pH of 0 after slaughter (Table 6). For meat colors, the results are shown in Table 7. Quadratic effects on CIE L*, a*, and b* values were observed as the dietary sulfur level increased up to 4% (quadratic; p<0.05).

Postmortem glycolysis and the conversion of glycogen into lactic acid influence meat pH differences [34]. Meat color is affected by many factors, including active myoglobin, oxygen concentration and enzymes in muscle, particularly changes in pH [35]. In particular, pork with a high level of oxymyglobin appears to have a higher redness than the level of metmyoglobin. As the pork was exposed to oxygen, oxygenation progressed from Mb to MbO_2_, and the surface color of the pork changed from purple to bright red. Jang et al [15] found that pH, redness and yellowness decreased but lightness increased as pigs were supplemented with dietary sulfur. Yang et al [36] also indicated that the pH value was significantly lower as well as CIE a* (redness) and CIE b* (yellowness) values and that the CIE L* (light reflection) value was higher when pigs were fed 0.1% dietary sulfur. In the present study, similar results were observed for pigs supplemented with dietary sulfur. However, Upadhaya et al [16] recently reported that pH and meat color were not affected by the addition of up to 0.4%.

## CONCLUSION

Inclusion of dietary nontoxic sulfur in growing-finishing pig diets up to 0.2% could improve growth performance, amino acid composition in hair and meat antioxidation capacity.

## Figures and Tables

**Table 1:** Formula and chemical composition of the experimental diet

Items	Experimental phases^1)^			

	Growing phase 1	Growing phase 2	Finishing phase 1	Finishing phase 2

	Basal diet			
Ingredient (%)				
Corn	70.18	75.64	70.18	84.49
Soybean meal-46%	21.76	16.64	21.76	8.44
Wheat bran	3.84	4.00	3.84	4.16
Tallow	1.59	1.33	1.59	0.79
L-Lysine-HCl, 55%	0.08	0.03	0.08	0.18
DL-Methionine, 90%	0.01	0.01	0.01	0.01
DCP	1.30	1.18	1.30	0.86
Limestone	0.74	0.67	0.74	0.58
Vit. Mix^2)^	0.10	0.10	0.10	0.10
Min. Mix^3)^	0.10	0.10	0.10	0.10
Salt	0.30	0.30	0.30	0.30
Sum	100.00	100.00	100.00	100.00
Chemical composition^4)^				
Metabolizable energy (kcal/kg)	3,300.00	3,300.00	3,300.00	3,300.00
Crude protein (%)	15.69	13.75	12.13	10.43
Total lysine (%)	0.83	0.67	0.66	0.52
Total methionine (%)	0.26	0.18	0.18	0.14
Calcium (%)	0.66	0.59	0.52	0.46
Total phosphorus (%)	0.56	0.52	0.47	0.43

1) The experimental phases were divided into four phases and every feeding phase was three weeks.

2) Provided per kg of diet: vitamins per kg of complete diet: vitamin A, 8,000 IU; vitamin D_3_, 1,800 IU; vitamin E, 40 IU; vitamin K_3_, 4 mg; thiamine, 2.00 mg; riboflavin, 7.0 mg; pantothenic acid, 20 mg; niacin, 50 mg; pyridoxine, 3 mg; d-biotin, 0.2 mg; folic acid, 1 mg; vitamin B_12_, 0.03 mg.

3) Provided per kg of diet: mineral per kg of complete diet: Se, 0.3 mg; I, 0.3 mg; Mn, 49 mg; Cu, 288 mg; Fe, 150 mg; Zn, 85 mg; Co, 2 mg.

4) Calculated value.

**Table 2:** Effect of dietary nontoxic sulfur on growth performance in growing-finishing pigs

Criteria	Treatment^1)^				SEM	p-value	
	
	Control	NTS 0.1	NTS 0.2	NTS 0.4		Linear	Quadratic
Body weight (kg)							
Initial	34.72	34.95	34.63	34.64	0.169	0.75	0.92
3 wk	46.75	44.97	44.06	44.75	0.524	0.30	0.24
6 wk	65.72	62.05	63.09	60.30	0.956	0.13	0.74
9 wk	82.00	83.00	83.80	75.04	1.263	0.04	0.08
12 wk	102.27	103.51	104.33	95.24	1.081	0.01	0.01
ADG (g)							
0 to 3 wk	572.61	477.26	449.47	481.52	27.151	0.36	0.24
4 to 6 wk	903.58	813.26	905.88	740.70	26.302	0.19	0.57
7 to 9 wk	775.28	997.66	986.11	701.89	38.560	0.17	0.01
10 to 12 wk	965.01	976.86	977.81	961.85	30.947	0.96	0.89
0 to 12 wk	804.12	816.26	829.82	721.49	13.027	0.01	0.01
ADFI (kg)							
0 to 3 wk	1.40	1.23	1.30	1.28	0.037	0.56	0.41
4 to 6 wk	1.93	1.87	1.84	1.76	0.070	0.48	0.98
7 to 9 wk	2.78	2.83	2.89	2.61	0.071	0.41	0.33
10 to 12 wk	3.29	3.17	3.17	3.20	0.093	0.83	0.73
0 to 12 wk	2.39	2.32	2.27	2.31	0.054	0.64	0.59
G:F ratio							
0 to 3 wk	0.41	0.39	0.37	0.38	0.024	0.71	0.73
4 to 6 wk	0.46	0.44	0.47	0.42	0.022	0.51	0.82
7 to 9 wk	0.28	0.35	0.34	0.27	0.011	0.32	0.02
10 to 12 wk	0.30	0.31	0.32	0.30	0.016	0.98	0.74
0 to 12 wk	0.34	0.35	0.37	0.31	0.008	0.25	0.12

SEM, standard error of the mean; ADG, average daily gain; ADFI, average daily feed intake; G:F, gain-to-feed.

1) Control, Corn-SBM-based diet; NTS 0.1, Control + nontoxic sulfur 0.1%; NTS 0.2, Control + nontoxic sulfur 0.2%; NTS 0.4, Control + nontoxic sulfur 0.4%.

**Table 3:** Effects of dietary nontoxic sulfur on serum IgA and IgG concentrations in growing-finishing pigs

Criteria	Treatment^1)^				SEM	p-value	
	
	Control	NTS 0.1	NTS 0.2	NTS 0.4		Linear	Quadratic
IgA (mg/mL)							
Initial	-------------------------------------------- 1.65 ------------------------------------------						
3 wk	1.80	2.39	2.46	2.06	0.153	0.77	0.17
6 wk	1.87	3.37	1.29	2.52	0.335	0.93	0.85
9 wk	1.51	1.58	1.73	1.76	0.123	0.52	0.84
12 wk	2.05	1.60	1.70	1.59	0.127	0.38	0.54
IgG (mg/mL)							
Initial	-------------------------------------------- 9.56 --------------------------------------------						
3 wk	10.00	10.00	9.58	10.00	0.073	0.83	0.15
6 wk	9.60	9.96	9.93	9.47	0.093	0.48	0.12
9 wk	10.00	9.54	9.79	9.27	0.113	0.12	0.98
12 wk	9.28	9.85	9.84	10.00	0.084	0.01	0.08

IgA, immunoglobulin A; IgG, immunoglobulin G; SEM, standard error of the mean.

1) Control, Corn-SBM-based diet; NTS 0.1, Control + nontoxic sulfur 0.1%; NTS 0.2, Control + nontoxic sulfur 0.2%; NTS 0.4, Control + nontoxic sulfur 0.4%.

**Table 4:** Effects of dietary nontoxic sulfur on hair cystine and methionine content in growing-finishing pigs

Criteria	Treatment^1)^				SEM	p-value	
	
	Control	NTS 0.1	NTS 0.2	NTS 0.4		Linear	Quadratic
Cystine (%)							
Initial	-------------------------------------------- 12.85 ---------------------------------------------						
Growing phase	12.93	12.79	13.63	13.64	0.365	0.48	0.89
Finishing phase	12.33	15.67	14.70	14.93	0.396	0.57	0.68
Methionine (%)							
Initial	-------------------------------------------- 0.29 --------------------------------------------						
Growing phase	0.32	0.33	0.32	0.30	0.008	0.08	0.06
Finishing phase	0.30	0.34	0.38	0.35	0.009	0.03	0.01

SEM, standard error of the mean.

1) Control, Corn-SBM-based diet; NTS 0.1, Control + nontoxic sulfur 0.1%; NTS 0.2, Control + nontoxic sulfur 0.2%; NTS 0.4, Control + nontoxic sulfur 0.4%.

**Table 5:** Effect of the dietary nontoxic sulfur on the physicochemical properties of pork in growing-finishing pigs

Criteria	Treatment^1)^				SEM	p-value	
	
	Control	NTS 0.1	NTS 0.2	NTS 0.4		Linear	Quadratic
Proximate analysis (%)							
Moisture	72.17	74.89	73.46	74.83	0.456	0.09	0.44
Crude protein	23.97	25.42	23.30	23.45	0.575	0.19	0.69
Crude fat	6.61	5.21	4.50	5.63	0.336	0.07	0.44
Crude ash	0.61	0.56	0.67	0.73	0.057	0.35	0.82
Physiochemical property							
Cooking loss (%)	28.67	26.61	27.15	26.08	0.559	0.29	0.68
Shear force	49.13	45.63	41.30	48.22	2.718	0.94	0.42
Water holding capacity	67.84	64.54	64.32	64.84	0.854	0.40	0.31
TBARS	0.105	0.081	0.054	0.089	0.005	0.34	0.01

SEM, standard error of the mean; TBARS, thiobarbituric acid reactive substances.

1) Control, Corn-SBM-based diet; NTS 0.1, Control + nontoxic sulfur 0.1%; NTS 0.2, Control + nontoxic sulfur 0.2%; NTS 0.4, Control + nontoxic sulfur 0.4%.

**Table 6:** Effect of dietary nontoxic sulfur on pH of pork in growing-finishing pigs

Criteria	Treatment^1)^				SEM	p-value	
	
	Control	NTS 0.1	NTS 0.2	NTS 0.4		Linear	Quadratic
pH							
0 h	6.21	6.05	5.77	5.76	0.067	0.04	0.30
3 h	5.71	5.71	5.58	5.71	0.075	0.92	0.51
6 h	5.60	5.87	5.65	5.78	0.062	0.53	0.68
12 h	5.65	5.87	5.72	5.90	0.049	0.19	0.88
24 h	5.76	5.82	5.89	5.83	0.047	0.65	0.55

SEM, standard error of the mean.

1) Control, Corn-SBM-based diet; NTS 0.1, Control + nontoxic sulfur 0.1%; NTS 0.2, Control + nontoxic sulfur 0.2%; NTS 0.4, Control + nontoxic sulfur 0.4%.

**Table 7:** Effect of dietary nontoxic sulfur on meat color of pork in growing-finishing pigs

Criteria	Treatment^1)^				SEM	p-value	
	
	Control	NTS 0.1	NTS 0.2	NTS 0.4		Linear	Quadratic
CIE value, L*							
0 h	19.56	35.25	31.80	24.61	1.971	0.79	0.01
3 h	29.76	30.05	35.66	30.03	1.163	0.76	0.10
6 h	23.25	23.20	23.21	20.00	1.749	0.60	0.78
12 h	26.43	21.65	24.13	32.64	1.552	0.12	0.13
24 h	19.62	33.52	26.28	28.47	1.721	0.29	0.15
CIE value, a*							
0 h	7.06	2.73	3.60	4.98	0.516	0.39	0.01
3 h	4.37	4.55	3.03	3.56	0.307	0.28	0.50
6 h	6.00	6.03	6.32	6.29	0.336	0.77	0.90
12 h	5.14	7.11	4.99	3.59	0.460	0.13	0.29
24 h	6.10	4.41	6.22	4.13	0.361	0.21	0.49
CIE value, b*							
0 h	12.26	9.32	11.19	13.03	0.439	0.10	0.02
3 h	11.01	11.28	9.50	10.18	0.356	0.35	0.53
6 h	8.34	11.69	12.04	11.70	0.514	0.08	0.07
12 h	10.91	11.99	10.09	9.93	0.359	0.16	0.77
24 h	11.21	9.91	10.97	9.98	0.376	0.42	0.92

SEM, standard error of the mean.

1) Control, Corn-SBM-based diet; NTS 0.1, Control + nontoxic sulfur 0.1%; NTS 0.2, Control + nontoxic sulfur 0.2%; NTS 0.4, Control + nontoxic sulfur 0.4%.
